# *Campylobacter* positivity and public health risks in live bird markets in Busia, Kenya: A value chain analysis

**DOI:** 10.1111/tbed.14518

**Published:** 2022-03-30

**Authors:** Josephat Mbai, Samuel Njoroge, Mark Obonyo, Christina Otieno, Maurice Owiny, Eric M. Fèvre

**Affiliations:** ^1^ Kenya Field Epidemiology and Laboratory Training Program Kenyatta Hospital Grounds Nairobi Kenya; ^2^ International Livestock Research Institute Nairobi Kenya; ^3^ Government of Makueni County Makueni Kenya; ^4^ Kenya Medical Research Institute KEMRI Nairobi Kenya; ^5^ School of Public Health Moi University Kesses Kenya; ^6^ Institute of Infection Veterinary and Ecological Sciences University of Liverpool Liverpool UK

**Keywords:** *Campylobacter*, Kenya, live bird market, poultry, public health

## Abstract

Live bird markets (LBMs) provide integral hubs for 95% of poultry produced for food. Surveillance systems in LBMs serving smallholder farmers in sub‐saharan Africa are often non‐functional, and data about public health risks and emerging pathogens are lacking. Studies in Kenya have reported 29–44% *Campylobacter* prevalence in poultry. We analysed such LBMs in Kenya for likely transmission of *Campylobacter* from poultry to humans. We conducted a cross‐sectional survey among 186 live poultry traders (LPTs) in 14 LBMs in a region with widespread backyard poultry systems. A pretested structured questionnaire was administered to all LPTs having regular contacts with poultry to gather market data and risk information on campylobacteriosis. *Campylobacter* was detected in individual cloacal cultures and identified through PCR. The median score obtained from the outcome of risk assessment dichotomized respondents into high and low risk categories. We performed logistic regression at 95% confidence interval (CI) to compare market characteristics and *Campylobacter* positivity to risk categories to identify LBM‐associated public health risks. Markets had a median of 13 traders, and mean age of 46.3 ± 13.7 years. Majority 162/186 (87.1%) were males. Market behavioural processes by LPTs varied: Only 58.6% LPTs held bird species separate; onsite slaughter (38.7%); encountered sick‐bird (93%) and dead‐bird (83%) amidst limited health inspection (31.2%). *Campylobacter* positivity in live birds was 43/112 (38.4%, 95% CI: 29.4–48.1). Risk information on campylobacteriosis was low 41/114 (36%, 95% CI: 27.2–45.5). Sanitary risks were related to accumulation of litter (adjusted prevalence odds ratio [aPOR]: 19.67, 95% CI: 3.01–128.52). Accessing hand‐wash facilities (aPOR: .32, 95% CI: .13–.78) and access to information (aPOR: .24, 95% CI: .09–.61) were protective. Sanitary risks were related to poor hygiene. LBMs could be central surveillance sites for *Campylobacter*. Public health authorities/actors should consider appropriate targeting to improve sanitary measures and *Campylobacter* control strategies.

## 1 INTRODUCTION

Poultry farming is an important component of the agricultural sector worldwide, providing food, nutrition and income (Mottet et al., 2017). The global poultry sector is projected to grow substantially at 24% to reach 131,255 metric tons by 2025 (Carron et al., 2017; Mottet et al., 2017). This growth is catalysed by urbanization, increasing populations and consumer taste (Food and Agriculture Organization [FAO], 2011; Okello et al., 2010). Poultry production is of vital importance in improving livelihoods of rural populations, but without a policy framework, it is likely the poorest smallholders who make up a large proportion of producers in many low‐ and middle‐income countries (LMIC) will be outclassed by the well‐resourced commercial operations from overall economic growth and transformation of market structures (Aklilu et al., 2007; Chaiban et al., 2020; Okello et al., 2010).

The poultry sector in Kenya contributes about 30% of agricultural gross domestic product (GDP) (Abubakar et al., 2019; Okeno et al., 2012). The consumption of poultry in Kenya is predicted to reach 164.6 metric tonnes by 2030 (Chaiban et al., 2020). Kenya's poultry population is estimated at 37 million birds at any given time, of which about 74−80% are raised in backyard settings (Abubakar et al., 2019; MOLFD, 2012; Okello et al., 2010). Backyard systems are unspecialized, raising on average 13–50 birds on low inputs and outputs to provide for home consumption and possibly raise cash without targeting specific markets. The sale of most backyard poultry occurs in either general food markets or stalls. In urban centres, the live poultry traders (LPTs) may confine birds in cages and stalls, while in rural areas the birds may be tied on open ground (McCarron et al., 2015; Molia et al., 2016).

Poultry value chain studies in Kenya have examined productivity and challenges within poultry systems to improve on performance and profitability (Okello et al., 2010; Okeno et al., 2012). Value chains analysis involves the mapping and description of the production‐supply, commercial and institutional environment in which businesses operate to supply goods to consumers (Carron et al., 2017; Okello et al., 2010; Rushton, 2011). Studies of trading networks may markedly recognize markets involved in the perpetuation of infection and be focus of control measures, seasonal fluctuations in volumes traded consistent with risk of transmission (Van Kerkhove et al., 2009) and the role of traders in potential transmission from village to village during collection and spread through market network (Tiensin et al., 2009).

Poultry are recognized asymptomatic carriers of several important human pathogens including *Campylobacter, Salmonella, Escherichia coli* and highly pathogenic avian influenza (HPAI) (Magalhães et al., 2010). In many LMICs, up to 95% of birds produced for food are marketed live or as freshly slaughtered in live bird markets (LBMs) (Cardona et al., 2009; Sayeed et al., 2017; Van Kerkhove et al., 2009). Several LBM‐based surveillance studies in Egypt, Bangladesh, Cambodia and China concluded the collective topographies of diverse ecological origins, close contact of various bird species, keeping birds on floors and inadequate hygiene measures from cleaning and disinfection, lack of an all‐in, all‐out management and longer than a day stay in LBMs promoted local transmission and genetic reassortment of pathogens (Abdelwhab et al., 2010; Fasina et al., 2016; Kayali et al., 2014; Martin et al., 2011). The detailed comprehension of interactions, interdependence of trade‐related structures and patterns of mobility in LBM can explain disease emergence and spread (Jones et al., 2016). Such an understanding of epidemiological and market behavioural processes is necessary to manage the diseases under such circumstances by developing appropriate mitigation on identified risk pathways and public health risks.

*Campylobacter* spp. is an emerging foodborne pathogen with significant threats to public health and substantial economic losses worldwide (Havelaar et al., 2015). Globally, it causes an estimated >95 million foodborne illnesses and >21,000 deaths annually (Kaakoush et al., 2015; Larsen et al., 2014; Thomas et al., 2020). Poultry are important reservoirs of human infection, and 90% of the human cases are caused by *Campylobacter jejuni* (Carron et al., 2018; Chlebicz & Śliżewska, 2018; Kaakoush et al., 2015). Human campylobacteriosis may be asymptomatic or cause inflammatory diarrhoeas, which could be blood stained in 30% of children associated with abdominal pain, fever, nausea and sometimes vomiting (Chlebicz & Śliżewska, 2018; Ravel et al., 2016). Serious sequelae include Guillain‐Barre’ syndrome (GBS), an autoimmune‐driven damage of human nerves (Chlebicz & Śliżewska, 2018; Kaakoush et al., 2015).

In many LMICs, surveillance for *Campylobacter* seldom exists in humans and poultry, and data pertaining the organism's presence, risk factors and impacts are rare (Asuming‐Bediako et al., 2019; Carron et al., 2018; World Health Organization, 2013). Large disparities ranging from 2 to 100% in prevalence of *Campylobacter* in chicken across different countries are reported (Meunier et al., 2016), and when amalgamated at a global level, leave global assessments of *Campylobacter* burden severely lacking in data from sub Saharan Africa. The European Food Safety Authority reports a mean *Campylobacter* prevalence of 70% in primary broiler production (Meunier et al., 2016), while a prevalence study for *Campylobacter* from 171 poultry premises and 53 retail traders in Nairobi, Kenya, reported a range of 33–44% for broiler and indigenous chicken farms, respectively, 60 and 64% for retailers (Carron et al., 2018). Poultry markets are constituent of the food chain, but the public health risks and emerging pathogens have not been fully elaborated in much of East Africa. These markets also lack policy guidelines to support the market development and food safety measures. This value chain study aimed at analysing LBMs to allow for better understanding of the public health risks of *Campylobacter* infection and detail possible entry points for intervention using Busia county as a case study. This multidimensional approach, linking value chain mapping and description of the wider market topologies, is in‐line with the FAO's (2011) recommendation to promote value chain analysis in animal diseases risk management.

## 2 MATERIALS AND METHODS

### 2.1 Study area

**FIGURE 1:**
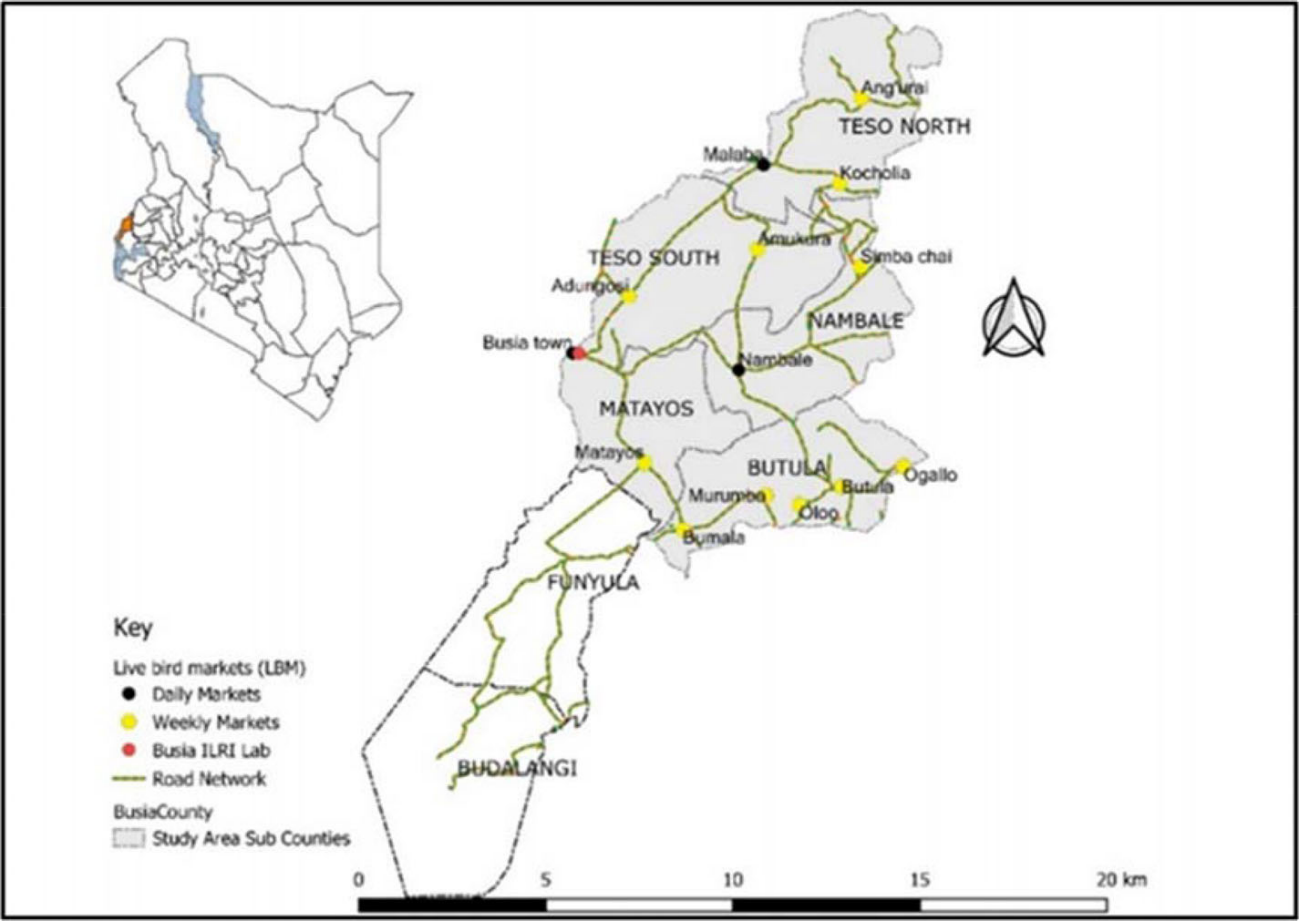
Map of Kenya showing Busia County and study sites (LBMs) in subcounties of Matayos, Butula, Nambale and Teso South & North (*Source*: https://africaopendata.org/dataset/kenya‐counties‐shapefile, 2019)

This study was conducted in Busia county, western Kenya in the Lake Victoria basin region on the border with Uganda (Figure 1). Busia county lies between latitude 0° 45 north and longitude 34° 25 east. Busia county is a site with a trading network for the supply of live birds mainly from backyard settings to the rest of Kenya (McCarron et al., 2015). Poultry production is typically extensive, estimated at 1.4 million birds and characterized by dualism of local and improved breeds. The majority of villages have a certain day each week for selling foodstuffs such as cereals, legumes and live birds in community markets.

### 2.2 Study design

We conducted a LBM‐based cross‐sectional survey from July to October 2018.

### 2.3 Study population

This study consisted of all LPTs having regular contacts with poultry. An LPT was defined as a trader with occupation requiring transportation of live poultry, selling live poultry or slaughtering of poultry for customers and comprised of sellers and middlemen. A seller would have a stall/cage at the market, while the middleman had none. In instances where the middleman also doubled as market seller, the respondent was asked which role they identified with most closely. We included LPT who had worked in the selected LBMs for at least six months prior to the survey, aged above 18 years old and willing to participate in the study. Eligible poultry species for obtaining *Campylobacter* faecal samples included chickens, ducks, geese, guinea fowl and turkeys. Those who bought poultry for home consumption and non‐designated LBM sites with less than three poultry stalls were excluded.

### 2.4 Sampling strategy

A minimum sample size of 170 LPTs was determined using 95% confidence level and 5% margin of error with a considered proportion of 87.3% LPTs who never separated birds by species (Kirunda et al., 2015). The formula of Thrusfield (2005) was used. It was estimated 12 traders operated within a market cluster (Molia et al., 2016) and therefore required 14 (170/12) LBMs to achieve the desired sample size. Multistage sampling strategy was used to recruit participants into the study. The primary sampling unit was the LBM, defined as a site where live birds are traded daily or on designated days either at an isolated location or at a location shared with a wider food market. Initially, five subcounties in Busia (Figure 1) were purposely selected, since they host most poultry trading centres and are major routes for the inter‐county poultry trade (McCarron et al., 2015). We selected LBMs based on random sampling without stratification from a total of 23 poultry markets listed by veterinary authorities and collaborated by revenue authorities in the five subcounties. During weekly market days, we enlisted all eligible LPTs using a census method. Arrangements were made with the market survey assistant for return visits to all eligible LBMs to enlist all LPTs. We considered poultry, usually about 20 held by one LPT to constitute a flock. To determine *Campylobacter* positivity, we randomly obtained individual cloacal faecal samples from one bird per flock held by the LPT.

### 2.5 Data collection

**FIGURE 2:**
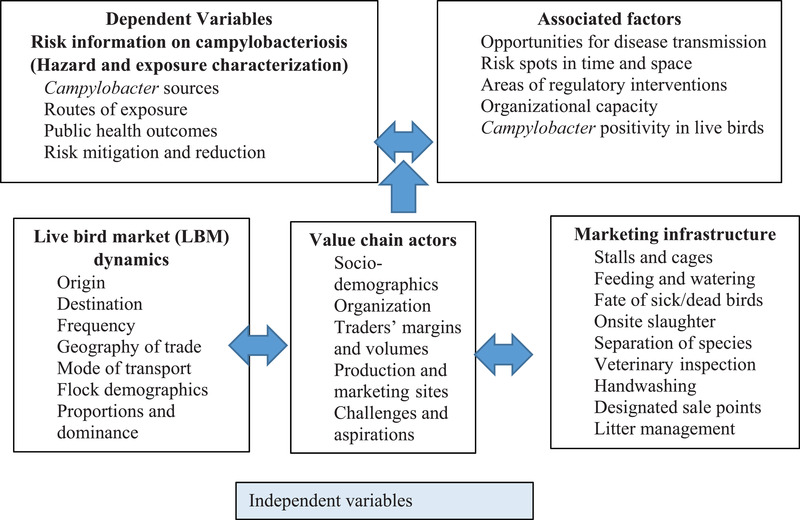
Conceptual framework – Value chain frameworks and public health risks in live bird markets, Busia County, 2018; adapted from Rushton, 2011

Using an interviewer‐administered pretested structured questionnaire, market survey data were collected (Conceptual framework, Figure 2) under five major headings: (i) Identity checklist; being date of interview, market name, locality geocodes and LPT unique identifier, (ii) poultry traders’ demographics; including gender, age, category (seller or middlemen), educational attainment, marital status, and years of experience of trading, (iii) market structure and operations; recruitment and management of LBMs, payment of levies, record keeping, bird species traded, suppliers of poultry, frequency of market (weekly or daily), number and prices of each species sold in a week, LPT weekly income, seasonality of trade and market outlets for poultry, (iv) market hygiene and biosecurity status; production system at source, mode of transporting poultry to the market, health inspection on arrival, feeding and watering, fate of unsold poultry, onsite slaughter, management of sick birds, fate of dead birds, cleaning of stalls and cages, accumulation of litter and use of protective clothing and (v) market governance and risk information; awareness and membership to market association, challenges and constraints in poultry trade and sources of poultry information. Using a rating response (disagree, do not know, no comment, agree and likely), seven item‐statements for knowledge on risk characterization were framed independently to identify sources of *Campylobacter*, routes of exposure, public health outcomes in susceptible populations and the risk reduction measures (Table 1).

**TABLE 1:** Responses to risk assessment of campylobacteriosis among poultry traders in live bird markets in Busia County, Kenya, 2018 (*n* = 186)

Criteria	Risk statement	Agree	Likely	No comment	Do not know	Disagree
*Campylobacter* sources	Poultry may host harmful bacteria in gastrointestinal tract without showing signs of sickness	76 (40.9)	39 (21.0)	13 (2.0)	35 (18.8)	23 (12.4)
	Process of slaughtering of live birds could possibly contaminate the carcass with harmful bacteria	87 (46.8)	40 (21.5)	4 (2.2)	32 (17.2)	23 (12.4)
Exposure assessment	Movement of poultry for trade can cause exchange and spread of poultry diseases	127 (68.3)	22 (11.8)	7 (3.8)	19 (10.2)	11 (5.9)
	Traders hands can become contaminated and cause ingestion of bacteria upon eating at the workplace	37 (19.9)	57 (30.7)	3 (1.6)	63 (33.8)	26 (14.0)
Public health outcomes	Consumption of contaminated chicken can cause gastroenteritis and diarrhoeal illness in susceptible persons	13 (7.0)	73 (39.3)	55 (29.6)	21 (11.3)	24 (12.9)
Risk reduction	Cleaning/disinfection of trading areas can reduce disease risks to other poultry and persons coming to LBMs	61 (32.8)	45 (24.2)	18 (9.7)	20 (10.8)	42 (22.6)
	Traders have realized government support through agribusiness and capacity building	6 (3.2)	11 (5.9)	4 (2.2)	8 (4.3)	157 (84.4)

*Abbreviation*: LBM, live bird market.

#### 2.5.1 Bacteriological testing for estimation of live bird *Campylobacter* positivity

One bird per poultry held by each LPT was randomly selected, restrained and cleaned for any obvious dirt around the outside of the cloaca with disposable wipes. A sterile swab moistened by dipping in Preston enrichment medium was placed inside the cloacal opening against the internal surface of the mucosa and rotated two to three times before being withdrawn. Using scissors dipped in 70% alcohol, the stem was cut at the brim of the bottle to allow tight closure. The media vial was then labelled with a unique epidemiological identifier, placed in an insulated chilled cool box packed with ice packs (5°C) and transported immediately within 5 hours of collection to the International Livestock Research Institute (ILRI) Busia laboratory for immediate processing. The procedure was repeated for the next poultry held and every participating LPT for every market cluster visited. At the laboratory, each cloacal faecal sample was tested for *Campylobacter* by culture and identification method described elsewhere (Wikler, 2017). Each universal bottle was opened aseptically and the homogenized faecal sample filtered through membrane filters onto modified charcoal cefoperazone deoxycholate (mCCD) agar prepared as described (Biesta‐Peters et al., 2019). The culture was incubated under microaerobic conditions at 42°C for 48 hours in anaerobic jar supplied with a gas‐generating kit (Campygen sachets, Oxoid). Subsequently, the mCCD agar plates were inspected for growth of round grey‐white or flat creamy colonies presumed *Campylobacter* spp. matched to previously validated characteristics of *Campylobacter genus* from pure colonies.

#### 2.5.2 Molecular identification of *Campylobacter* spp

Genomic DNA was both extracted and purified from bacteria cells presumed to be *Campylobacter* spp. using a commercially available kit supplied by Norgen Biotech (Norgen Biotek Corp., Thorold, ON, Canada) following the manufacturer's recommendations. The DNA samples were stored at −40°C. Conventional polymerase chain reaction (PCR) was carried out on all the presumed positive samples. The Linton PCR primers C412F/CampR2 were used to amplify a 906 bp fragment of the 16S ribosomal RNA gene (GenBank accession No. Z29326.1) as previously described (Gonzalez et al., 2000). The PCR amplification reactions contained 3 μl genomic DNA, 0.5 μl each of the forward and reverse primers and 12.5 μl of Taq PCR Master Mix (Qiagen) in a final reaction volume of 28 μl. After denaturation at 95°C for 10 min, amplification cycles were performed in four stages: stage one: 25 cycles of 95°C for 30 s, 58°C for 30 s and 72°C for 30 s for seven cycles; stage two: 95°C for 30 s, 56°C for 30 s and 72°C for 30 s for seven cycles; stage three: 95°C for 30 s, 55°C for 30 s and 72°C for 30 s for seven cycles and stage four: 95°C for 30 s, 54°C for 30 s and 72°C for 30 s for four cycles followed by 72°C for 10 min and cooling to 10°C. The PCR products were loaded on 1.2% (w/v) Hi‐Standard Agarose gel (AGTC Bioproducts Limited, Hessle, UK) in 1× tris‐Boric‐EDTA and stained with 0.5 μg/ml SafeWhite Nucleic Acid Stain (NBS Biologicals, Cambridgeshire, UK). Electrophoresis was carried out for 40 min at 190 V. The bands were visualized in UV trans‐illuminator and digitally photographed. Linton PCR‐positive samples were further screened to determine if they are *Campylobacter coli* using primers to amplify a 364 bp region of the glutamyl aminotransferase gene, gatB. The primers Jej‐3 and Jej‐4 were used to screen for *C. jejuni* as previously described (Rosenquist et al., 2007).

### 2.6 Data management and statistical analysis

Market survey data and laboratory test results were entered, cleaned and analysed using Microsoft Excel (Microsoft Office, Seattle, USA), Epi‐Info™ version 7 (CDC, USA) and QGIS software. For the participants’ responses; ‘disagreed’ and ‘do not know’ to a statement on risk information were scored zero [0], while ‘agreed’ and ‘likely response’ to the statement earned one [1] score. Responses of ‘no comment’ were excluded from the analysis. We performed descriptive statistics for LBM variables. The means with their standard deviations (SD), medians with their interquartile range (IQR) for continuous variables and proportions for categorical variables were calculated. Awareness of risk information was determined by summation of individual LPT outcomes for each statement to give a total score per respondent. We calculated the median score from the aggregate of scores attained by all respondents. Respondents with scores ≤ the median score were categorized as ‘high risk’, while scores > the median as ‘low risk’. We assessed the significance of risk information scores for market demographics using the Mann‐Whitney test at *p* < .05. *Campylobacter* positivity was determined for cloacal faecal samples according to market centre and bird species sampled. The proportion of *Campylobacter*‐positive birds was assessed for associated market conditions expressed as prevalence odd ratios (PORs) at *p* < .05.

In both bivariate and binary logistic regression, the dichotomous categorization of risk characterization was used as a dependent variable. We assessed the strength of association between risk category and exploratory variables such as participant demographics and market characteristics. Variables associated with risk outcome in bivariate analysis with a *p* < .2, and/or theoretical importance of probability to the risk outcome were fitted into a multivariable logistic regression model. The final multivariable model was constructed using a backward elimination approach. Variables were reported in the best‐fit model based on statistical significance of *p* < .05, final likelihood ratio and results expressed as adjusted prevalence odds ratio (aPOR) with 95% CI and two‐tailed *p*‐values.

### 2.7 Ethical considerations

We obtained ethical approval to conduct the study from Institutional Research and Ethics Committee (IREC) of Moi University and Moi Teaching Referral Hospital (FAN: IREC 2058). Both administrative and veterinary authorities in Busia county authorized access to LBMs. A local resident was identified and retained as market survey assistant in every LBM as a key person of contact to obtain access to market and gain trust of market sellers. Prior to the market survey and sampling, we organized market visits and held discussions with LPTs to explain the study objectives, participants’ rights and obtain informed written consent. Names of respondents were not recorded on specimen labels.

## 3 RESULTS

### 3.1 Respondent demographic characteristics

We enrolled and interviewed all 186 eligible poultry sellers. Five LPTs declined interviews because they felt they had participated in similar surveys in the past. Mean age was 46.3 ± 13.7 years, while 70.4% were aged between 25 and 54 years. The majority (87.1 %) of LPTs were males, while most (74.7%) had attained primary school level of education. The LPTs median years of experience in trading was 10 years (IQR = 5–16).

### 3.2 The LBMs value chain framework

#### 3.2.1 Participation in LBMs

Markets had a median of 13 traders (IQR = 8–18). Almost two‐thirds (68.3%) were market sellers and 31.7% middlemen. Although the majority (85.5%) of LPTs reported an absence of formal licensing, almost all LPTs (97.3%) paid market levies to the county authority. Of the 89.3% LPTs reporting awareness of a market poultry association, 82.5% were association members. The poultry association was organized at each subcounty, comprising an average of 27 members.

#### 3.2.2 Poultry market supplies and operations

**FIGURE 3:**
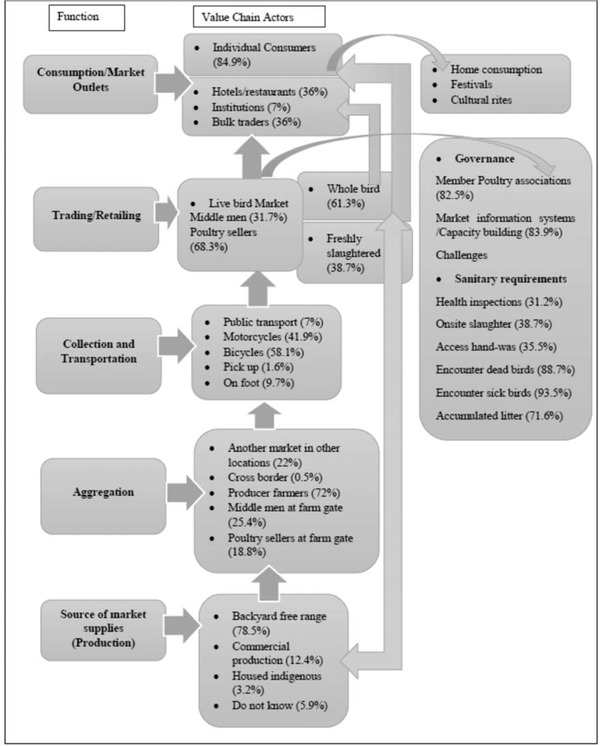
Value chain mapping of live bird market, Busia County, 2018

Backyard free range accounted for 78.5%, while 12.4% were commercial breeds transacted via producer farmers at 72.2%; another market in other location, 22%; cross‐border trade with Uganda, 0.5%; middlemen, 25.4% and sellers themselves at 18.8% collecting from community villages (Figure 3). Poultry were mainly transported using bicycles (58.1%) and motorcycles (41.9%). Markets operated either daily (21.4%) in Malaba, Busia and Nambale or on market days twice a week in other LBMs trading in various species and ages. The LPT median (IQR) weekly turnover was: chicken 20 (10–40), ducks 4 (2–9), turkeys 2 (1–3) and geese 3 (2–10) and earned weekly gross income of Ksh 1300 per trader (IQR = 500–3000) (1 USD = Ksh 100), although only 31.7% of LPTs were likely to keep transaction records.

#### 3.2.3 Outlets for market stock

Customers for poultry held in LBMs were: 84.9% individual consumers, 36.6% restaurants and hotels, 36% aggregation for bulk traders and 7% to both public and private institutions (Figure 3). Further, 88.7% of LPTs reported selling breeding stock to farmers. The peak periods for poultry sales were related to traditional circumcision ceremonies in August, national public holidays and religious festivals in April and December.

### 3.3 Challenges of the poultry trade

These were poultry morbidities and mortalities at 60.8%; inadequacy of business capital, 36%; trading in stolen poultry, 32.8%; taxation, unfair competition and levies, 19.4%; bad debts to creditors, 12.4%; unstable markets, 28% and poor market infrastructure, 28%. Except for Malaba, which had a designated trading area, birds in other markets were sold in cages or tied on the ground in a section of the food market.

### 3.4 Poultry information and sources

Only 16.1% of LPTs reported a lack of capacity building. Among the 83.9% who reported having benefited from capacity building around their LPT role, when asked the source of information, slightly less than a half (48.1%) had received information from fellow traders, 32.1% from animal health professionals, 25.6% from workshops and seminars, 25% through radio and television, 8.3% school curriculum, 3.2% from public health officers and 1.3% from information, education and communication (IEC) materials.

### 3.5 Estimation of *Campylobacter* positivity in live birds

Owing to laboratory capacity and time constraints, only eight (57.1%) of LBMs and 112 LPTs were surveyed. We obtained 46 (41.1%) growth on mCCD agar of grey‐white or flat creamy colonies presumed positive of *Campylobacter* spp. Further molecular diagnosis through PCR identified 43 (93.5%) for *Campylobacter*, 906 bp fragment of the 16S ribosomal RNA gene. Overall, *Campylobacter* positivity in live birds was 43/112 (38.4%, 95% CI: 29.4–48.1) and speciated: *C. genus* (4.7%); *C. coli* (11.6%) and *C. jejuni* (83.7%) (Table 2). Cloacal faecal samples from guinea fowls showed no growth of *Campylobacter*. Of the eight LBMs surveyed, *Campylobacter* positivity was highest in Nambale (27.9%) and Busia town (18.6%) (Table 2). *Campylobacter* positivity in live birds was potentially associated with: feeding birds awaiting sales (POR 2.31, *p* = .478), providing drinking water (POR 2, *p* = .365), sick birds in the flock (POR 1.95, *p* = .708) and encountering death in flock (POR 1.46, *p* = .763), although these were not statistically significant.

**TABLE 2:** *Campylobacter* positivity in live birds, Busia, Kenya 2018

Variable	Frequency, *n*	Proportion, %
Poultry species sampled *n* = 112		
** ** Chicken	104	92.9
** ** Ducks	5	4.5
** ** Guinea fowl	3	2.7
*Campylobacter* colonies on culture, *n* = 112		
** ** mCCD presumed‐positive colonies	46	41.1
** ** mCCD no growth/negative	66	58.9
PCR results *n* = 46		
** ** Positive for *Campylobacter* genome	43	93.5
** ** Negative for *Campylobacter* genome	3	6.5
** ** Overall positivity *n* = 112	43	38.4
Strain differentiation *n* = 43		
** ** *Campylobacter genus*	2	4.7
** ** *Campylobacter coli*	5	11.6
***Campylobacter jejuni***	**36**	83.7
Poultry species positivity *n* = 43		
** ** Chicken	40	93
** ** Ducks	3	7
LBM positivity, *n* = 43		
** ** Bumula	6	14.9
** ** Ogallo	1	2.3
** ** Busia town	8	18.6
** ** Matayos	2	4.7
** ** Nambale	12	27.9
** ** Adongosi	3	7
** ** Amukura	1	2.3
** ** Simba chai	2	4.7

*Abbreviations*: mCCD, modified charcoal cefoperazone deoxycholate agar; PCR, polymerase chain reaction.

### 3.6 Identification of LBM‐associated sanitary risks

#### 3.6.1 Risk characterization

The risk assessment measure was related to poultry sellers’ knowledge on campylobacteriosis based on seven‐item statements (Table 1). Seventy‐two respondents recorded a ‘no comment’ to one or more of the risk statements and were excluded from analysis (*n* = 114). Only 41 (36%; 95% CI: 27.2–45.5) LPTs attained rating > the median score of four. These were categorized as ‘low risk’, while 73 (64%; 95% CI: 54.5–72.8) were categorized as ‘high risk’. None of the LBMs in Busia had a designated slaughter area, 38.7% of LPTs reported onsite slaughter of birds for their customers on demand and had limited use of personal protective equipment (PPE) at 7.5%, while a third (31.2%) reported an encounter with veterinary health inspection teams.

#### 3.6.2 Market demographics

Variations in risk assessment scores were statistically significant for LPT category (Mann–Whitney [M–W] test = 3.68, *p* = .054), market centres (M–W test = 36.57, *p* = .005) and frequency of markets (M–W test = 9.08, *p* = .002). Market demographics of sex, marital status, age group, religion and level of education were not statistically significant for risk information scores.

#### 3.6.3 Market structure and operations

Markets that operated on weekly market days had lesser odds of public health risks than daily markets (POR = .47, 95% CI: .14–1.44), while non‐association members showed a higher risk compared to poultry association members (POR = 1.5, 95% CI: .45–5.82). Keeping trading records (POR = .90, 95% CI: .41–2.01) had potential for sanitary risks reduction though not statistically significant (Table 3).

**TABLE 3:** Predictors of public health risks in live bird markets in Busia county, Kenya, 2018 (*n* = 114)

Variable	Total	Risk status		Bivariate analysis			Multivariable analysis		
	*n*	High, *n* (%)	Low, *n* (%)	POR	95% CI	*p*	aPOR	95% CI	*p*
Sex									
Male	98	61 (62.2)	37 (37.8)	0.55	0.12–2	.481^a^	0.6	0.15–3.19	.642
Female	16	12 (75)	4 (25)	Ref					
LPT category									
Seller	76	53 (69.7)	23 (30.3)	2.07	0.93–4.63	.073	0.71	0.22–2.26	.557
Middlemen	38	20 (52.6)	18 (47.4)	Ref					
LBM frequency									
Weekly market day	89	54 (60.7)	35 (39.3)	0.47	0.14–1.44	.237^a^	0.36	0.11–1.21	.09
Daily	25	19 (76)	6 (24.0)	Ref					
Kept records									
Yes	40	25 (62.5)	15 (32.5)	0.9	0.41–2.01	.802	0.68	0.26–1.80	.441
No	74	48 (64.9)	26 (35.1)	Ref					
Provided water									
Yes	97	62 (63.9)	35 (36.1)	0.97	0.27–3.15	1^a^			
No	17	11 (64.7)	6 (35.3)						
Provided feeding									
Yes	99	65 (65.7)	34 (34.3)	1.67	0.47–5.77	.394	1.38	0.22–8.81	.735
No	15	8 (53.3)	7 (46.7)	Ref					
Member of association (*n* = 105)									
No	19	14 (73.7)	5 (26.3)	1.5	0.45–5.82	.595	6.2	0.93–41.16	.055
Yes	86	56 (65.1)	30 (34.9)	Ref					
Health inspection									
No	76	52 (71.2)	24 (31.6)	1.75	0.79–3.91	.167			
Yes	38	21 (55.1)	17 (44.8)	Ref					
Species separation									
Yes	70	45 (64.3)	25 (35.7)	1.02	0.47–2.26	.944	0.6	0.2–1.62	.315
No	44	28 (63.6)	16 (36.4)	Ref					
Accumulation of litter (*n* = 107)									
Yes	72	50 (69.4)	22 (30.6)	1.52	0.65–3.52	.332	19.7	3.01–128.52	.002
No	35	21 (60)	14 (40)	Ref					
Onsite slaughter									
Yes	48	32 (66.7)	16 (33.3)	1.22	0.56–2.66	.618	1.7	0.53–5.41	.371
No	66	41 (62.1)	25 (37.9)	Ref					
Handwashing access									
Yes	39	20 (51.3)	19 (48.7)	0.44	0.20–0.97	.041	0.32	0.13–0.78	.001
No	75	53 (70.6)	22 (29.3)	Ref					
*Campylobacter* status (*n* = 70)									
Positive	36	22 (61.1)	14 (38.9)	0.97	0.37–2.55	.955	0.52	0.16–1.64	.264
Negative	34	21 (61.8)	13 (38.2)	Ref					
Fate of sick birds									
Quick sale	38	20 (52.6)	18 (47.4)	0.74	0.26–2.06	.564			
Self‐medicate	22	16 (72.7)	6 (27.3)	1.78	0.44–7.47	.542^a^			
Slaughter for self	24	15 (62.5)	9 (37.5)	1.11	0.35–3.51	.857			
Kill for disposal	14	12 (85.7)	2 (14.3)	4.67	0.76–49.43	.191^a^	1.74	0.12–24.73	.684
Segregate	25	15 (60)	10 (40)	Ref					
Fate of dead birds									
Consume oneself	7	3 (42.9)	4 (57.1)	0.53	0.06–3.7	.721^a^	1.09	0.06–18.8	.95
Throw to municipal waste	15	10 (66.7)	5 (33.3)	1.4	0.34–6.38	.841^a^			
Thrown into open pit	58	39 (67.2)	19 (32.8)	1.44	0.6–3.45	.416			
Bury	34	20 (58.8)	14 (41.2)	Ref					
Information sources									
Livestock professionals	35	17 (46.6)	18 (51.4)	0.37	0.12–1.2	.163	0.24	0.09–0.61	.004
Radio	21	12 (51.1)	9 (42.9)	0.53	0.15–1.92	.519	0.66	0.16–2.58	.551
Fellow traders	52	34 (65.4)	18 (34.6)	0.76	0.25–2.28	.824	0.73	0.22–2.34	.59
School curriculum	5	4 (80)	1 (20)	1.6	0.12–91.7	1^a^			
Workshop/seminars	26	13 (50)	13 (50)	0.4	0.11–1.35	.234	0.75	0.18–3.13	.698
Friends and family	21	15 (71.4)	6 (28.6)	Ref					

*Note*: Final – 2* likelihood = 127.93, Cases included = 114, Likelihood ratio = 49.49, *p* < .001.

*Abbreviations*: aPOR, adjusted prevalence odds ratio; CI, confidence interval; LBM, live bird markets; LPT, live poultry traders; POR, prevalence odds ratio; Ref, reference group.

^a^Fisher's exact values.

#### 3.6.4 Market biosecurity practices

**FIGURE 4:**
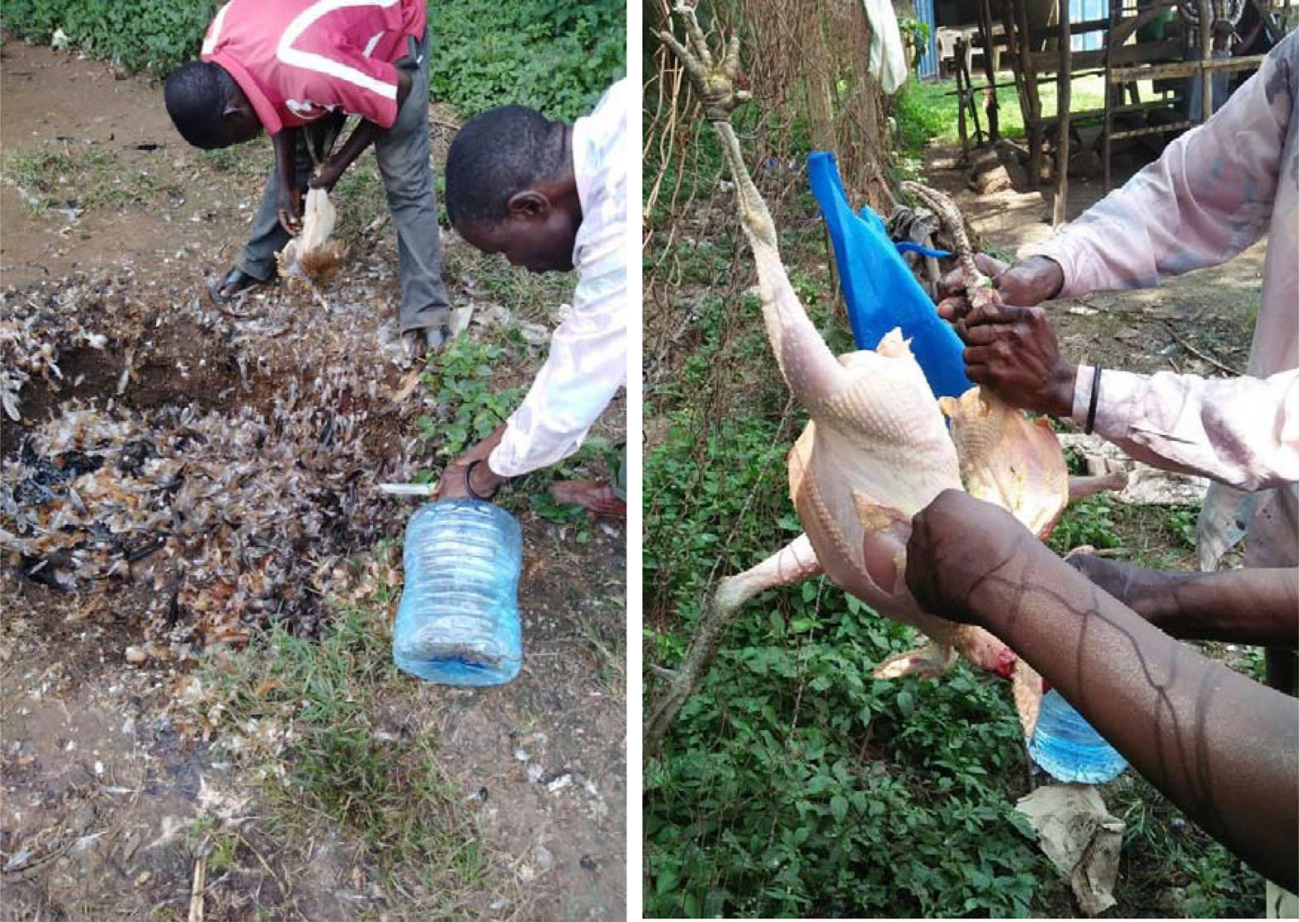
Poor hygiene practices among poultry sellers in live bird markets in Busia County, Kenya, 2018

Potential sanitary risks arose from absent veterinary inspection, POR = 1.75 (.79–3.91), onsite slaughter, POR = 1.22 (.7–2.69), self‐medication of sick birds, POR = 1.78 (.44–7.47) as well as killing sick birds for disposal, POR = 4.67 (.76–49.43). Others were throwing dead birds to municipal waste (POR = 1.4 [.6–3.45]) or open pit (POR = 1.44 [.6–3.45]) and presence of *Campylobacter*‐positive live birds (POR = .97 [.37–2.55]) (Table 3). Of the 94.6% traders with stalls and cages, 71.6% had visibly accumulated litter in their wares, and litter was also noticed to pile in market sites (Figure 4). In bivariate analysis, traders having accumulated litter in stalls and cages had 1.5 times higher odds of public health risks compared to traders with clean cages (POR = 1.52 [.65–3.52]).

### 3.7 Multivariable analysis for public health risks in LBMs

In the final model, accumulation of litter (adjusted POR, 19.67, 95% CI: 3.01–128.52) independently increased exposure to public health risks. Accessing hand‐wash facilities (aPOR: .32, 95% CI: .13–.78) and access to information (aPOR: .24, 95% CI: .09–.61) remained significantly protective. Other variables in the model did not attain statistical significance. Weekly markets had 36% lesser odds of risk compared to daily markets (aPOR: .36, 95% CI:.11–1.21). Non‐association members had 6.2 times higher odds of sanitary risks compared to association members (aPOR: 6.2, 95% CI: .93–41.16) and onsite slaughter potentially increased exposure to public health risks (aPOR = 1.7, 95% CI: .55–5.41) (Table 3).

## 4 DISCUSSION

This study examined LBMs in a region typical of smallholder poultry farming for public health risks associated with likely transmission of *Campylobacter* spp. from poultry to humans. *Campylobacter* positivity and sanitary risks are highlighted particularly in the context of backyard poultry settings where the burden and epidemiology of *Campylobacter* remain scanty (Asuming‐Bediako et al., 2019; Carron et al., 2018). Market behavioural processes occasioned sanitary risk hotspots related to waste and litter management, minimal health inspection visits and *Campylobacter*‐positive live birds. The study details possible entry points for intervention such as market information systems and hygiene measures. This multidimensional approach, linking value chain mapping and description of the wider market topologies, is in‐line with the FAO's (2011) recommendation to promote value chain analysis in animal diseases’ risk management.

### 4.1 Market demographics

The dominance of males in these LBMs contrasts Egyptian traditional markets, which are largely operated by women vendors (Abdelwhab et al., 2010). Socio‐cultural factors have been reported to influence gender participation in markets in other livestock production systems (Aklilu et al., 2007). The higher male participation could be associated with access to financial resources, ability to take risks and access to market information (Aklilu et al., 2007). The understanding of the gender differences in Busia could contribute to identifying opportunities for women to participate and bargain power in the overall economic growth of these markets. The mean age of 46 ± 13.7 years implied that the energetic, enterprising and active productive age were major participants in LBMs. A risk management study of 84 poultry farmers in Imo, Nigeria reported this age category was likely to enjoy enduring capacity for innovations and risk bearing (Iheke et al., 2016). A contextual understanding why participation of this age group increases could contribute to realization of opportunities to improve market access and benefits. The LBMs were largely driven by LPTs (75%) with a primary level of education. Parallel proportion of LPTs’ educational levels 74–84% in Uganda (Kirunda et al., 2015) and 79% in Bangladesh (Sayeed et al., 2017) has been reported. The learning attainment may depict traders’ literacy and numerical skills and possible entrepreneurship skills gained over the years. This could impact positively for decision making, since the practical knowledge acquired over time could be useful in overcoming certain inherent deficits and customer tastes in the market environment (Aklilu et al., 2007; Elelu, 2017). An Egyptian study by Kayali et al. (2014) also reported that the educational status limits their options for gainful engagement. He understandably argues that any interventions being made in the LBMs should be appraised for implications that seem to threaten their economic activity (Kayali et al., 2014).

### 4.2 Mechanisms for supply and retention of birds

Poultry trade was largely an individual undertaking without formal marketing linkages. This contrasts organized retail poultry shops in Egypt (Abdelwhab et al., 2010) and well‐structured retail and wholesale bird markets in Chittagong metropolis of Bangladesh offering both live and dressed poultry (Sayeed et al., 2017). The informal and low marketing allows them to exert their independence and control over their own sales but exposes them to possible economic instabilities of inadequate finances, ravages of poultry diseases and make access to agribusiness services difficult (Aklilu et al., 2007).

The LBMs connected backyard production systems and commercial producers to traders and consumers. Even though the region's agriculture is intensifying, backyard poultry farming dominates in western Kenya (Chaiban et al., 2020, Okeno et al., 2012). These poultry systems are characteristic of inadequate maintenance of biosecurity and have been associated with outbreaks of poultry diseases such as salmonellosis, Newcastle and HPAI (Okello et al., 2010; Okeno et al., 2012), which are likely to compromise biosecurity of LBMs and health of consumers (Carron et al., 2017). Increase in volume of poultry deliveries to LBMs mirrors Egypt and Ethiopian poultry markets which coincided with secular and religious festivals (Abdelwhab et al., 2010; Aklilu et al., 2007; ElMasry et al., 2017). The bidirectional movement of birds in and out of markets and farms reported has previously been described in Kenya and Cambodia poultry movement networks (Carron et al., 2017; Van Kerkhove et al., 2009). Similarly, Kayali et al. (2014) postulate that these could be farmers acquiring stock to target maturation to coincide with the times of the year for premium sales and satisfy annual trade patterns. These movements and seasonal fluctuations in volumes traded could be consistent with risk of transmission (Van Kerkhove et al., 2009) as is the role of traders in potential transmission from village to village during collection and spread through market network (Tiensin et al., 2009).

### 4.3 Value chain actor relationships

Poultry market associations were present in the LBMs. Membership‐to‐market associations promote equity, are source of credit, structure the marketing of products and likely reduce transaction costs for obtaining market information (Carron et al., 2017; Okello et al., 2010). The challenges reported had implications on LBM performance, and similar constraints have been identified in other livestock production systems (Mutua et al., 2017; Okello et al., 2010). Our study agrees with other studies recommendations for formulation of policies to improve traders’ use and access to agribusiness services to alleviate impacts of these challenges (Carron et al., 2017; Okello et al., 2010).

#### 4.3.1 *Campylobacter* positivity

This study demonstrates the presence of *Campylobacter* spp. in live birds in LBMs (38.4%). Busia County is one of the trading networks for supply of live birds to the rest of Kenya (McCarron et al., 2015). Further, as the birds sampled originated in small holdings, it is likely that home slaughter and consumption of birds from the same flocks results in household level exposures. The majority of customers in LBMs were individual public consumers (85%), restaurant and hotels (37%), highlighting the risk of *Campylobacter* infection from LBMs poultry intended for consumption. *Campylobacter* positivity in live birds in Busia is within the 33–44% prevalence for broiler and indigenous poultry, respectively, but lower than 66% prevalence at retail markets of chicken meat system in Nairobi, Kenya (Carron et al., 2018). It contrasts with the 22.5% *Campylobacter* prevalence for broiler farms within a much more intensive system mentioned elsewhere (Abubakar et al., 2019) and 92% overall prevalence for broiler farms in selected counties in Kenya. The paucity of data from similar studies in an East African context raises some difficulties in comparative analysis (Asuming‐Bediako et al., 2019; Carron et al., 2018), but our findings provide essential baseline information for this kind of production system in the Lake Victoria basin. Differences in positivity could be attributed to the number of live birds sampled, to a difference in size of market flocks, or a difference in sampling unit and testing methods.

Higher positivity of *Campylobacter* was detected for live birds in daily markets than in weekly markets. Similar observations were seen during HPAI surveillance in LBMs in Bangladesh and Egypt (55% and 36.6% AI incidence, respectively) possibly attributed to transmission dynamics and survival (Abdelwhab et al., 2010; Hassan et al., 2018). Daily markets are likely to have more slaughtering points and multiple species confined in spaces resulting in transmission within the market setting, when compared to weekly markets (Cardona et al., 2009; Kirunda et al., 2015). The close contact and mixing of birds from different markets and sources and limited epidemiological data are obstacles for identification of source infection in these markets, where more work is clearly required. However, LBMs may be ideal central point surveillance sites for emergent variants of *Campylobacter*, where access to households may be difficult or pose logistical challenges (Falzon et al., 2019).

### 4.4 Public health risks

Consistent with risk factors for infection with *Campylobacter*, public health risks were related to poor hygiene practices and missing food safety inspections. Onsite slaughtering of poultry with limited access to water supplies poses a risk of contamination of carcasses with intestinal bacteria and subsequently human infections (Figure 4). These biosecurity shortfalls may have been occasioned by limited access to information on LBM‐associated public health risks from reliable sources (Table 3). In such market settings, Elelu (2017) suggests the utilization of several illustrations and verbal communication tools such as onsite posters or broadcasts would impact more with traders than formal settings.

The poor biosecurity standards and risky practices including onsite slaughter, absent health inspection, self‐medication of sick birds and consumption and/or disposal of sick and dead birds in poor market facilities resonated with the weak governance and regulatory framework. Similar weak government controls have been reported in the Nairobi chicken supply system (Carron et al., 2017). The persistence of these market risks could mean the market disease burden is poorly understood. The low use of PPE was a major barrier to adoption of precautionary measures. Familiarity with endemic poultry diseases may have occasioned lesser perceived risk of infection among LPTs and therefore less protective actions despite regular contacts with poultry comparable to risk perception studies in south East Asia (Fielding et al., 2009). The accumulation of litter in market sites more so in stalls and cages posed the greatest risk of poultry transmissible infections (aPOR 19.67). Studies have reported *Campylobacter* spp. to remain viable in litter for up to six days (Abubakar et al., 2019). Market cleaning on rest days and disinfection have been reported to reduce prevalence of infectious diseases in Egypt and Bangladesh LBMs (ElMasry et al., 2017; Kim et al., 2018). The majority of LBMs were largely idle in between weekly market days and the downtime could accord these markets regular cleaning and disinfection.

This study compliments other studies that have evaluated LBMs on public health risks posed to traders and customers coming to these markets (Asuming‐Bediako et al., 2019; Kirunda et al., 2015; Larsen et al., 2014; McCarron et al., 2015; Molia et al., 2016). Foodborne diseases associated with poultry are widespread and represent significant threats to public health and substantial economic losses (Havelaar et al., 2015; Mottet et al., 2017). The detailed comprehension of interactions, interdependence of trade‐related structures and patterns of mobility in LBM can explain disease emergence and spread (Jones et al., 2016). This could possibly guide the institution of control measures and communication strategy to reduce potential pathogen spread in the LBMs and assure public health.

### 4.5 Limitations

Selection bias could have emanated from failure to contact some of the LPTs during our visits to LBMs. The return visits to enlist all LPTs ensured sample representativeness, high response rate and minimized selection bias. Asking traders to recollect their trading practices with scanty record keeping might have introduced recall bias to the data collected. Pretesting carefully structured questionnaire and training of interviewers served to reduce this bias. The modest sample size for cloacal faecal samples arose from constraints of time and resources and may have introduced bias in some descriptive measures, but all samples remained randomly allocated. It is likely the estimated positivity captured diversities in live birds from diverse production systems, hygiene and biosecurity status, factors previously described to be associated with *Campylobacter*‐positive flocks. The positivity data and sample distribution are LBM based, which limit wider implications of the study to poultry systems with different dynamics. Nonetheless, the study results present a useful foundation for sanitary risk management in LBMs.

## 5 Conclusions and recommendations

This study generates an understanding of informal LBMs in smallholder farming environments to reveal *Campylobacter* positivity in live birds and associated public health risks in East Africa where data are scarce. The LBMs were unstructured, displaying multi‐species from different poultry production systems. *Campylobacter*‐positive flocks may contaminate the environment and serve as a reservoir for transmission and spread of the pathogen to other poultry and market actors as well as consumers of poultry from these markets. Poultry sellers had knowledge gaps regarding public health risks associated with contacts with poultry and had minimal use of PPEs. The value chain framework allows better understanding of the public health risk of *Campylobacter* infection and provides a framework for appropriate targeting to improve sanitary measures and *Campylobacter* control strategies. The LBMs could provide an ideal site for surveillance of emerging pathogens and development of context‐specific prevention and control models.

## CONFLICT OF INTEREST

The authors declare no conflict of interest.

## Data Availability

The data sets used and analysed for this study are provided as supplementary data (Mbai et al., 2022).
